# SARS-CoV-2 infection in dogs, dog owners, dog breeders, and veterinarians in the Ashanti and Greater Accra regions of Ghana—zoonotic implications

**DOI:** 10.3389/fvets.2026.1897559

**Published:** 2026-08-13

**Authors:** Esther Amemor, Raphael Deladem Folitse, Achim Hoerauf, Marijo Parcina, Manuel Ritter, Vitus Burimuah, Bertha Asuamah Yeboah, Benjamin Obukowho Emikpe

**Affiliations:** 1 Department of Clinical Studies, School of Veterinary Medicine, Kwame Nkrumah University of Science and Technology (KNUST), Kumasi, Ghana; 2 German-West African Centre for Global Health and Pandemic Prevention (G-WAC), Partner Site Kumasi, Kumasi, Ghana; 3 Department of Pathobiology, School of Veterinary Medicine, Kwame Nkrumah University of Science and Technology (KNUST), Kumasi, Ghana; 4 University of Bonn, University Hospital Bonn, Institute for Medical Microbiology, Immunology and Parasitology (IMMIP), Bonn, Germany; 5 German-West African Centre for Global Health and Pandemic Prevention (G-WAC), Partner Site Bonn, Bonn, Germany; 6 German Center for Infection Research (DZIF), Partner Site Bonn-Cologne, Bonn, Germany; 7 Department of Public Health and Epidemiology, School of Veterinary Medicine, Kwame Nkrumah University of Science and Technology (KNUST), Kumasi, Ghana

**Keywords:** COVID-19, dog breeders, dog owners, dogs, Ghana, SARS-CoV-2, veterinarians

## Abstract

**Background:**

After the COVID-19 pandemic, there is an increasing concern over the causative agent, *SARS-CoV-2*, mutating into a more virulent strain in animals and spilling over to humans, a potential threat of future pandemics. Anthropo-zoonotic transmissions of the virus can as well not be ruled out. The dynamics of spillover of *SARS-CoV-2*, particularly from humans to animals and has not been fully studied. Livestock and pet owners, as well as animal health workers, have been documented to be at greater risk of COVID-19 infection. To effectively control and prevent the COVID-19 disease, the virus needs to be adequately studied in humans, animals and the environment. There is limited information on COVID-19 infections in animals in Ghana.

**Methods:**

A cross-sectional study was carried out to ascertain the COVID-19 infection status in dogs, their owners, dog breeders, and veterinarians in Ghana, specifically in the Ashanti and Greater Accra regions. Blood samples and nasopharyngeal swabs were taken from dogs for serological and molecular analysis. Questionnaires on COVID-19 risk factors were administered. *SARS-CoV-2* antibodies in sera and viral RNA in swabs were detected using ELISA and RT- PCR, respectively.

**Results:**

It was observed that 2.3% (CI = 1%−5.3%) of the dogs tested positive on RT-PCR for *SARS-CoV-2*; 5.1% (CI = 2.4%−10.7%) of humans tested positive on PCR for *SARS-CoV-2*. A seroprevalence of 6.5% (CI = 3.9%−0.6%) of *SARS-CoV-2* was detected in dogs, and 99.1% (CI = 95.3%−99.8%) seropositivity was detected in humans. Close proximity to livestock was statistically associated with active SARS-CoV-2 infection in dogs (*p* = 0.004). In humans, a statistically significant association was found between infrequent mask use in public and a positive *SARS-CoV-2* infection (*p* = 0.009).

**Conclusion:**

This study provides evidence of natural SARS-CoV-2 infection in dogs in Ghana, among the first such indications in the sub-Saharan African region. Close proximity to livestock was associated with infection in dogs and inconsistent mask use with infection in humans, though the cross-sectional design precludes determination of transmission direction, and these associations should be regarded as hypothesis-generating. Longitudinal studies with larger samples and viral sequencing will clarify SARS-CoV-2 transmission dynamics among humans, animals, and environment.

## 1 Introduction

Coronaviruses (CoVs) are enveloped, positive-sense, single-stranded RNA viruses of the order *Nidovirales*, family *Coronaviridae*, and subfamily *Orthocoronavirinae* (1). The subfamily comprises four genera namely *Alphacoronavirus, Betacoronavirus, Gammacoronavirus*, and *Deltacoronavirus* of which α- and β-CoVs primarily infect mammals. Seven coronaviruses are known to infect humans, the most recent being severe acute respiratory syndrome coronavirus 2 (SARS-CoV-2) (2). While HCoV-229E, HCoV-OC43, HCoV-NL63, and HCoV-HKU1 cause mild upper respiratory infections, SARS-CoV, MERS-CoV, and SARS-CoV-2 are associated with more severe respiratory illness. SARS-CoV and MERS-CoV produced major outbreaks following their emergence in 2002 and 2012, respectively. In December 2019, clusters of pneumonia of unknown cause were reported in Wuhan, China; the causative agent, initially designated 2019-nCoV, was later identified as a novel coronavirus and the disease named COVID-19 (2, 3).

Most SARS-CoV-2 infections (approximately 81%) are mild, presenting with upper respiratory symptoms such as nasal congestion, sore throat, dry cough, low-grade fever, headache, myalgia, and fatigue (4). Moderate cases involve cough, dyspnoea, and tachypnoea, whereas severe disease may progress to pneumonia, acute respiratory distress syndrome, sepsis, or septic shock (4, 5).

The SARS-CoV-2 outbreak represents the third instance of a coronavirus breaching the species barrier, after SARS and MERS. These zoonotic strains have drawn considerable attention owing to their pathogenicity and broad host adaptability (6). Early in the pandemic, SARS-CoV-2 infection was recorded in numerous domestic and wild animal species, including cats, dogs, tigers, minks, lions, and deer (7). A wide range of additional hosts has since been identified as susceptible, including raccoons, hamsters, ferrets, pangolins, gorillas, cattle, and various non-human primates (7, 8).

Although data on specific risk factors for SARS-CoV-2 infection in dogs and cats remain limited, seropositivity has been linked to close-contact behaviors such as co-sleeping with infected owners and kissing pets (9). Given the virus's widespread human circulation and pets' limited exposure to other animals, companion-animal infections are presumed to arise primarily from human-to-animal transmission, although intra-household transmission among pets cannot be excluded. The first confirmed companion-animal case was a Pomeranian dog in Hong Kong in February 2020, followed by a domestic cat in March 2020, both tested after their owners were diagnosed with COVID-19 (10). High pathogen exposure has also been documented among livestock farmers, farmhands, abattoir workers, and animal caregivers (11), and persons in close contact with animals may be at risk of zoonotic spillover in both directions (12, 13).

Despite growing global evidence of SARS-CoV-2 infection in companion animals, data from sub-Saharan Africa remain scarce, and studies to that have documented the infection status of dogs or their associated human contacts in Ghana remains scarce. This paucity of regional information limits understanding of the virus's animal-human interface in the country and underscores the need for locally generated evidence. This study was therefore conducted to determine the COVID-19 infection status among dogs, their owners, dog breeders, and veterinarians in Ghana, specifically in the Ashanti and Greater Accra regions.

## 2 Methods

### 2.1 Study design

A cross-sectional study was carried out to determine whether dogs, their owners, dog breeders, and veterinarians had SARS-CoV-2 or its antibodies, using ELISA and RT-PCR. Structured questionnaires were used to gather information on potential risk factors that could influence virus circulation among the target populations. This study was conducted between January 2024 and June 2025.

### 2.2 Sampling

#### 2.2.1 Sampling technique

A purposive sampling approach based on known COVID-19 exposure risk factors was employed in this study, as described by Rahman et al. (14), in which the technique was used to identify participants who were more likely to have experienced COVID-19-related risks or disruptions. Dogs, dog owners, dog breeders, and veterinarians with frequent human–animal interaction or increased likelihood of exposure to SARS-CoV-2 were purposely selected from veterinary clinics, breeding facilities, and households within the study areas. This approach was adopted to maximize the likelihood of detecting SARS-CoV-2 infection and exposure among populations considered to be at relatively higher risk.

#### 2.2.2 Target population

Dogs, their owners, dog breeders, and veterinarians in contact with the target dogs.

#### 2.2.3 Inclusion criteria

Dogs aged three months and older together with their owners, breeders, and the veterinarians in contact with them were included in this study.

#### 2.2.4 Sample size calculation

We used the following formula to arrive at a sample size of 150 humans and 246 dogs, arrived; N = t^2^xp (1-p)/m^2^, with m = margin of error (5% or 0.05), n = sample size, t = level of confidence (95% whose value type is 1.96), and p = estimated proportion of the population that exhibits the trait under study (0.5 if no value is known) (15).

### 2.3 Sample collection

From each dog, 5 ml of blood was taken from the cephalic vein with a sterile 23-gauge needle and a 5 ml syringe. Nasopharyngeal swabs were also taken from the dogs by inserting a sterile swab stick into the nasopharynx. Swabs were kept in Viral transport medium (VTM) in cryotubes and stored at −80 degrees Celsius. Centrifugation was used to separate sera from blood samples, and the samples were subsequently chilled to −80 degrees Celsius.

From human participants, five (5 ml) of blood was taken from the cephalic vein with a sterile 23-gauge needle and a 5 ml syringe. Nasopharyngeal swabs were also taken from dog owners, dog breeders, and veterinarians by inserting a sterile swab stick into the nasopharynx. Swabs were kept in Viral transport medium (VTM) in cryotubes and stored at −80 degrees Celsius. Samples of blood were centrifuged, and sera were separated and subsequently stored at −80 degrees Celsius.

Pre-tested structured questionnaires were used to collect data on the participants' demographics and risk factors for COVID-19 infection.

### 2.4 Laboratory diagnosis

Molecular detection of SARS-CoV-2 RNA in nasopharyngeal swab samples collected from dogs and humans was performed using the Detection Kit for 2019-nCoV RNA (EUROIMMUN Medizinische Labordiagnostika AG, Germany; reported specificity 99.6%). All procedures were conducted in accordance with the manufacturer's instructions with minor laboratory adaptations where necessary.

#### 2.4.1 Nucleic acid extraction

Prior to extraction, the vacuum-sealed 96-well extraction plate supplied with the kit was removed from its packaging and centrifuged at 500 rpm for 1 min to ensure that the magnetic beads and reagents settled at the bottom of the wells. The aluminum sealing film was carefully removed immediately before use to minimize disruption of the reaction wells and avoid reagent spillage. Sample tubes were mixed thoroughly using a laboratory shaker for approximately 1–2 min to ensure homogeneity before processing.

For automated nucleic acid extraction, 200 μL of each prepared sample was dispensed into the designated wells of the extraction plate and loaded into the automated nucleic acid extraction system. Extraction was subsequently carried out according to the manufacturer's recommended protocol. At the completion of the extraction cycle, eluates were transferred into sterile centrifuge tubes for downstream molecular analysis, while excess extracts were stored at −20 °C until further use.

#### 2.4.2 Detection of SARS-CoV-2 RNA by multiplex real-time RT-PCR fluorescence probing

Highly purified RNA was extracted from both test samples and negative controls following the manufacturer's recommended nucleic acid extraction procedures. The lyophilised positive control recombinant plasmid supplied with the kit was reconstituted using 50 μL of sterile purified water before use. For amplification, the RT-PCR reaction mixture was prepared by dissolving the lyophilised 2019-nCoV RT-PCR mix in 1 mL of sterile purified water. The mixture was gently agitated and allowed to stand for approximately 30–60 s until a clear solution was obtained. Thereafter, 20 μL of the prepared RT-PCR master mix was dispensed into each reaction tube. Subsequently, 5 μL of extracted nucleic acid from each sample was added to the designated reaction tubes.

Similarly, 5 μL of the reconstituted positive control and 5 μL of the extracted negative control template were added into their respective control tubes. The reaction tubes were vortexed for approximately 10 s and briefly centrifuged before being loaded into the fluorescence real-time PCR instrument. Sample positions and reaction parameters were configured according to the manufacturer's operating instructions. Amplification was performed under the following cycling conditions: reverse transcription at 50 °C for 5 min, initial denaturation at 95 °C for 2 min, followed by 42 cycles of denaturation at 95 °C for 5 s and annealing/extension at 55 °C for 35 s.

##### 2.4.2.1 Interpretation of results

Run validity was confirmed by acceptable amplification of the positive control within the designated fluorescence channels and the absence of amplification; or Ct values exceeding the defined threshold in the negative control. Runs failing these criteria were deemed invalid and repeated. Samples were interpreted as SARS-CoV-2 positive when at least two target genes (ORF1a/b, N gene, or S gene) yielded Ct values ≤ 37 with characteristic sigmoidal amplification curves. Samples in which all target genes were undetected were interpreted as negative. Where only a single target gene was amplified, the assay was repeated; persistent single-target detection prompted collection of a fresh sample. Amplification of the RNase P gene served as an internal control for sample adequacy and extraction efficiency. Failure of RNase P amplification was attributed to inadequate sampling, suboptimal transport conditions, or extraction failure, and necessitated repeat sample collection and re-testing.

#### 2.4.3 Anti-SARS-CoV-2 ELISA (IgG) assay

Detection of anti-SARS-CoV-2 IgG antibodies in human serum samples was performed using the EUROIMMUN Anti-SARS-CoV-2 ELISA (IgG) kit (Germany; reported specificity 99.6%). In addition, nucleocapsid protein (NCP) ELISA was used to differentiate infection-induced antibodies from vaccine-induced antibodies.

A total of 100 μL each of calibrators, positive controls, negative controls, and diluted serum samples were dispensed into the respective microplate wells according to the manufacturer's instructions. The plate was incubated at 37 °C for 60 min and subsequently washed three times using working-strength wash buffer. Thereafter, 100 μL of enzyme conjugate containing peroxidase-labeled anti-human IgG was added to each well and incubated for 30 min at room temperature. Following an additional washing step, 100 μL of chromogen/substrate solution was added and incubated for 30 min at room temperature in the dark. The reaction was terminated using 100 μL of stop solution, and optical density was measured photometrically at 450 nm using a reference wavelength between 620 and 650 nm.

##### 2.4.3.1 Test evaluation and interpretation

Results were assessed semi-quantitatively by computing the ratio of the extinction of the control or patient sample over the extinction of the calibrator, using the following formula:


$$\text{Ratio =}\frac{\text{Extinction of the control or patient sample}}{\text{Extinction of calibrator}}$$ (1)

The mean absorbance of duplicate wells was used for all calculations. Ratios < 0.8 were interpreted as negative; ratios ≥0.8 to < 1.1 as borderline; and ratios ≥1.1 as positive, in accordance with the manufacturer's guidelines.

#### 2.4.4 Anti-SARS-CoV-2 nucleocapsid protein (NCP) ELISA

Detection of anti-SARS-CoV-2 nucleocapsid protein (NCP) antibodies was performed using the EUROIMMUN Anti-SARS-CoV-2 NCP ELISA kit (Euroimmun AG, Lübeck, Germany) to distinguish natural infection-induced from vaccine-induced immune responses.

A total of 100 μL each of calibrators, positive controls, negative controls, and diluted serum samples were dispensed in duplicate into the designated microplate wells and incubated at 37 °C for 60 min. Wells were washed three times with working-strength wash buffer, after which 100 μL of peroxidase-labeled anti-human IgG conjugate was added and incubated for 30 min at room temperature. Following a further washing step, 100 μL of chromogen/substrate solution was added and incubated in the dark for 15 min at room temperature. The reaction was terminated by the addition of 100 μL of stop solution, and absorbance was measured at 450 nm with a reference wavelength of 620–650 nm using a microplate reader.

##### 2.4.4.1 Test evaluation and interpretation

Results were calculated and interpreted using the same ratio formula and cut-off thresholds described in Section 2.4.3.1 (negative: < 0.8; borderline: ≥0.8 to < 1.1; positive: ≥1.1). Mean absorbance values of duplicate determinations were used for all interpretations.

#### 2.4.5 Detection of *SARS-CoV-2* IgG in dog sera

SARS-CoV-2 antibodies in canine serum samples were detected using the Eradikit COVID-19 Multispecies Double Antigen ELISA Test (IN3 Diagnostic, Turin, Italy), a validated multispecies assay for the detection of total immunoglobulins against SARS-CoV-2. A total of 60 μL of serum dilution buffer was dispensed into each well, followed by the addition of negative controls, positive controls, and test samples into their respective wells. The plate was incubated at room temperature for 60 min with gentle agitation. Wells were washed three times with diluted wash buffer and blotted dry. Diluted enzyme conjugate was then added and incubated for 45 min at room temperature. Following a further washing step, 100 μL of substrate solution was added and incubated in the dark for 10 min at room temperature. The reaction was terminated by adding 100 μL of stop solution, and absorbance was measured at 450 nm.

##### 2.4.5.1 Results interpretation

Percentage reactivity (PR) was calculated for each sample as follows:


$$PR\;=\;\left[\frac{\left(OD_{sample}\;-\;OD_{NegC}\right)}{\left(OD_{PosC}\;-\;OD_{NegC}\right)}\right]\times \;100$$ (2)

where OD denotes the mean absorbance of duplicate wells. Samples with PR ≥20% were interpreted as positive; those with PR < 20% were interpreted as negative. Run validity required an optical density >1.0 for the positive control and a positive-to-negative control OD ratio >3.

#### 2.4.6 Data analysis

Data were entered into Microsoft Excel 2019, cleaned for completeness and consistency, and exported into IBM SPSS Statistics version 27 for analysis. Descriptive statistics were used to summarize demographic, clinical, exposure, and behavioral variables. Categorical variables were presented as frequencies and percentages, while SARS-CoV-2 seroprevalence and RT-PCR prevalence were calculated as proportions of positive samples among the total samples tested. Associations between SARS-CoV-2 test results and potential factors in dogs and humans were assessed using Chi-square test or Fisher's exact test where appropriate. Statistical significance was set at *p* < 0.05.

## 3 Results

### 3.1 Demographic characteristics of sampled dogs and humans

A total of 215 dogs were sampled. Most were female (56.7%), aged 1–5 years (67.4%), and kept under semi-enclosed management (57.7%). Mixed/local breeds and other specific breeds together made up the majority. Most dogs were vaccinated against core canine diseases (85.6%), and 18 (8.4%) lived in close proximity to livestock (Table 1).

**Table 1:** Dog demographic characteristics.

Demographic variable	Category	Number (%)
Total animals		215 (100)
Sex	Female (F)	122 (56.7)
	Male (M)	90 (41.9)
	Unknown/Not specified	3 (1.4)
Age (years)	< 1 year	53 (24.7)
	1–5 years	145 (67.4)
	5–10 years	9 (4.2)
	Unknown/Not specified	8 (3.7)
Breed	Boerboel	37 (17.2)
	German Shepherd	19 (8.8)
	Mixed/Local/Mongrel	66 (30.7)
	Other Specific Breeds	80 (37.2)
	Unknown/Not Specified	13 (6.0)
Management system	Semi-enclosed	124 (57.7)
	Enclosed	73 (34.0)
	Free roaming	12 (5.6)
	Unknown/Not specified	6 (2.8)
Presence of livestock nearby	Yes	18
	No	197
Vaccination status	Yes	184 (85.6)
	No	24 (11.2)
	Unknown/Not specified	7 (3.3)

A total of 117 participants were enrolled, mostly male (61.5%), aged 18–24 years (59.8%), and single (81.2%). Veterinary students formed the largest professional group (76.1%). Awareness of zoonotic diseases was high (94.0%), and most participants were vaccinated against COVID-19 (70.1%), predominantly with AstraZeneca (37.8% of those vaccinated) (Table 2).

**Table 2:** Human demographic data.

Demographic variable	Category	Number (%)
Total participants		117 (100)
Sex	Male (M)	72 (61.5)
	Female (F)	45 (38.5)
Age group	18–24 years	70 (59.8)
	25–34 years	33 (28.2)
	35–44 years	7 (6.0)
	45–54 years	4 (3.4)
	55 & above	3 (2.6)
Professional description	Veterinary student	89 (76.1)
	Veterinarian	17 (14.5)
	Para-veterinarian	6 (5.1)
	Dog owner/Other	5 (4.3)
Religion	Christian	115 (98.3)
	Others	2 (1.7)
Marital status	Single	95 (81.2)
	Married	18 (15.4)
	Cohabiting/Widowed	4 (3.4)
Awareness of zoonotic diseases	Yes	110 (94.0)
	No	7 (6.0)
Knowledge source	Lectures	79 (67.5)
	Media/Research/Reading	29 (24.8)
	Other/Not specified	9 (7.7)
Is brucellosis zoonotic?	Yes	104 (88.9)
	No/Not specified	13 (11.1)
Is COVID-19 zoonotic?	Yes	82 (70.1)
	No	29 (24.8)
	Not specified	6 (5.1)
COVID-19 vaccination status	Vaccinated (≥1 dose)	82 (70.1)
	Not Vaccinated	35 (29.9)
Vaccine type (if vaccinated)	AstraZeneca	31 (37.8% of vaccinated)
	Johnson & Johnson	24 (29.3% of vaccinated)
	Pfizer-BioNTech/Moderna/Others	27 (32.9% of vaccinated)

### 3.2 Seroprevalence of *SARS-CoV-2* in dogs and humans

**Figure 1:**
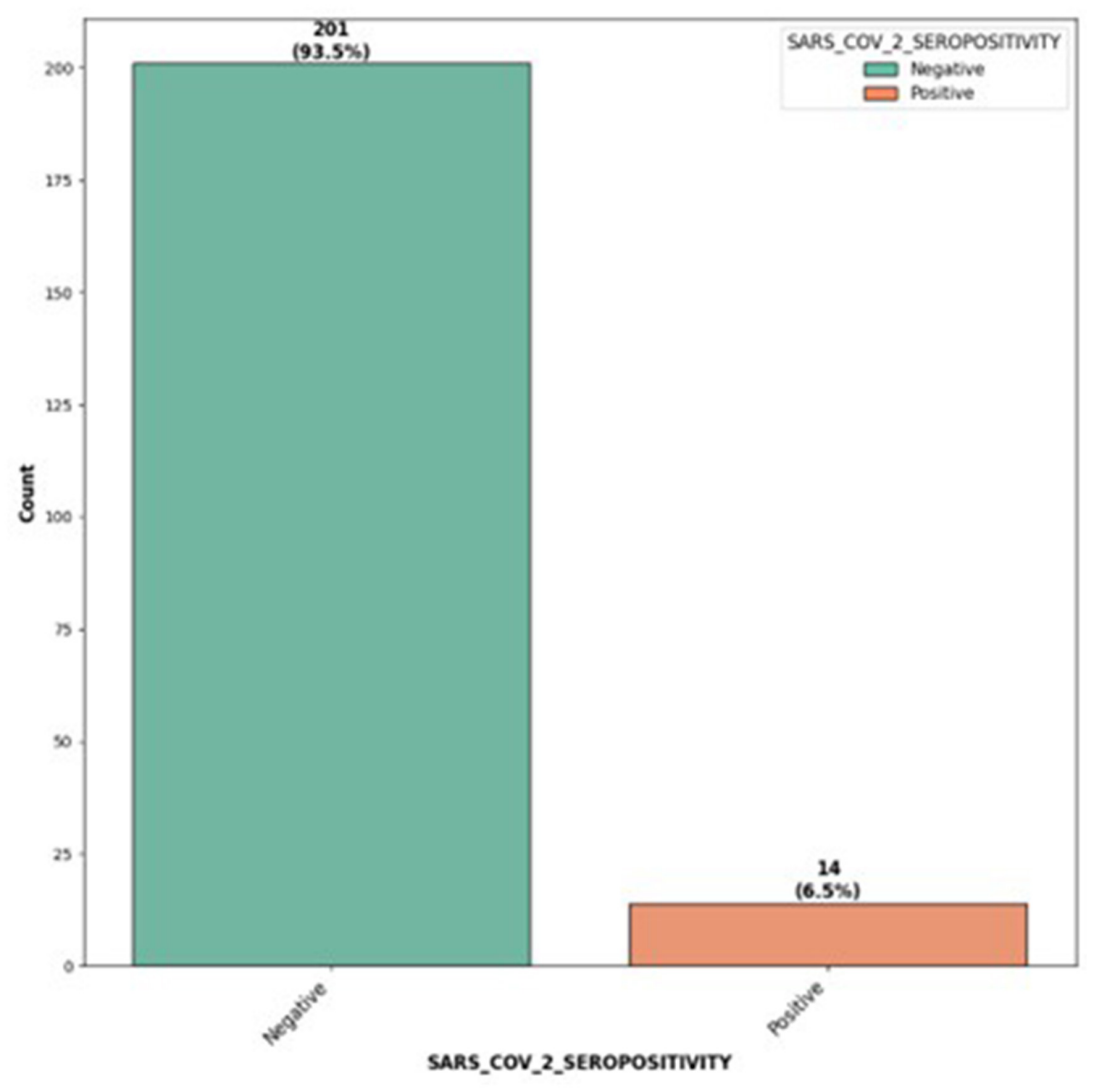
Seroprevalence of *SARS-CoV-2* in Dogs.

**Figure 2:**
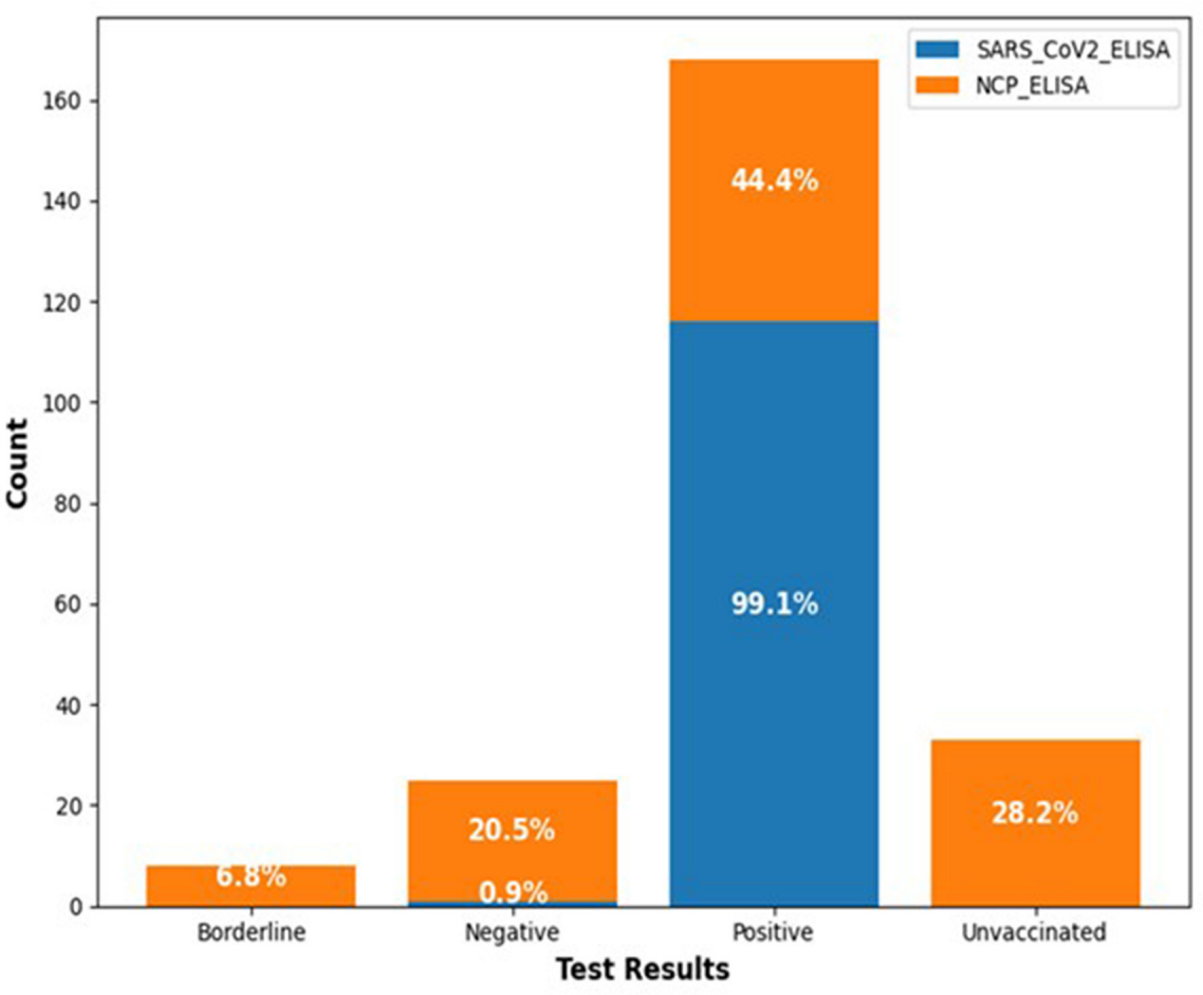
Differentiation of field-induced and vaccine-induced anti-*SARS-CoV-2* antibodies in human participants by NCP ELISA.

Figure 1 shows the seroprevalence of the dogs sampled using ELISA. Of 215 dogs sampled, 14 (6.5%; CI = 3.9%−10.6%) tested positive for *SARS-CoV-2*, and 201 (93.5%) were negative. Seroprevalence of *SARS-CoV-2* in human participants was 99.1% for both vaccinated and non-vaccinated. NCP ELISA was used to differentiate participants who tested positive for structural protein, indicating field-induced antibodies (44.4%) from those with vaccine-induced antibodies (Figure 2).

### 3.3 Prevalence of *SARS-CoV-2* in dogs by RT-PCR

**Figure 3:**
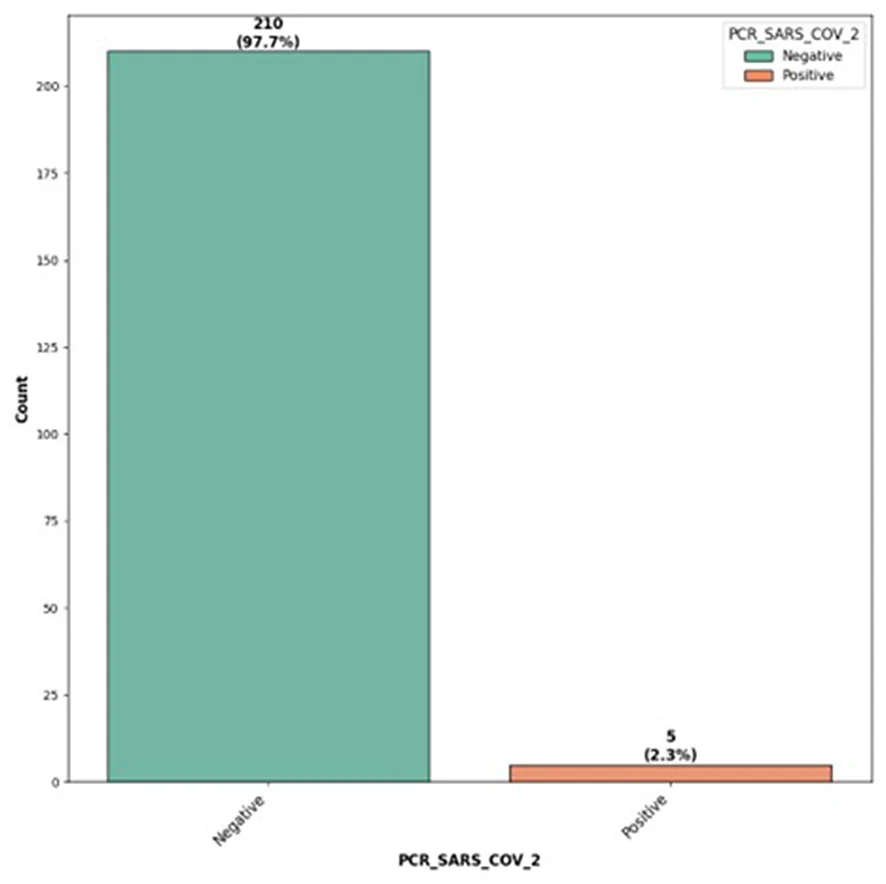
Prevalence of *SARS-CoV-2* in dogs by PCR.

**Figure 4:**
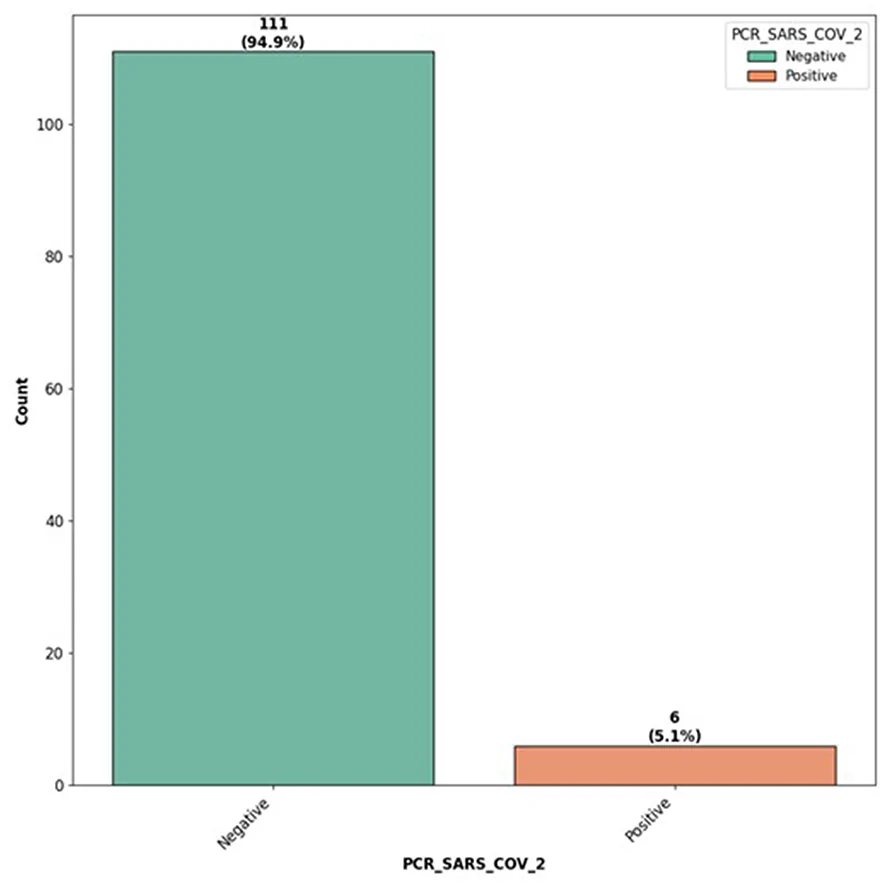
Prevalence of *SARS-CoV-2* in humans by PCR.

Figure 3 shows the occurrence of *SARS-CoV-2* in the sampled dogs using the RT-PCR test. Of the total of 215 dogs sampled, 5(2.3%; CI = 1.0%−5.3%) tested positive for *SARS-CoV-2* and 210 (97.7%) tested negative. Active infections of SARS-CoV-2 were detected in dog owners, dog breeders and Veterinarians (Figure 4).

### 3.4 Assessment of associated factors of *SARS-CoV-2*

Findings in Table 3 showed close proximity to livestock was significantly associated with SARS-CoV-2 PCR positivity in dogs (*p* = 0.004). No significant associations were detected between infection status and age, sex, management system, presence of neighboring dogs, vaccination status, frequency of interaction with other dogs, or contact with a COVID-19 patient (*p* > 0.05; Table 3).

**Table 3:** Association between demographic of dogs and *SARS-CoV-2* in dogs.

Variable	Categories	SAR-CoV-2		Total	Chi-square value	Fisher's exact test	*p*-value
		Negative	Positive				
If yes, any respiratory illness	No	74 (88.1%)	10 (11.9%)	84 (100.0%)	0.00	1.00
	Yes	0 (0.0%)	0 (0.0%)	0 (0.0%)		
Age	1–5	137 (65.6%)	9 (4.3%)	146 (69.9%)	5.586	0.061
	5–10	5 (2.4%)	2 (1.0%)	7 (3.3%)		
	< 1	53 (25.4%)	3 (1.4%)	56 (26.8%)		
Sex	Female	115 (54.2%)	8 (3.8%)	123 (58.0%)	1.039	1.00
	Male	83 (39.2%)	6 (2.8%)	89 (42.0%)		
Management system	Enclosed	93 (44.3%)	5 (2.4%)	98 (46.7%)	1.447	0.485
	Free Roaming	11 (5.2%)	0 (0.0%)	98 (46.7%)		
	Semi-enclosed	93 (44.3%)	8 (3.8%)	101 (48.1%)		
Presence of neighboring dogs	No	61 (29.3%)	3 (1.4%)	64 (30.8)	1.682	0.557
	Yes	133 (63.9%)	11 (5.3%)	144 (69.2%)		
Dog vaccinated	No	17 (8.2%)	0 (0.0%)	17 (8.2%)	inf	0.606
	Yes	178 (85.6%)	13 (6.2%)	191 (91.8%)		
Interaction with other dogs	Always	5 (3.1%)	0 (0.0%)	5 (3.1)	4.957	0.292
	Never	47 (29.0%)	1 (0.6%)	48 (29.6%)		
	Often	9 (5.6%)	0 (0.0%)	9 (5.6%)		
	Rarely	63 (38.9%)	3 (1.9%)	66 (40.7%)		
	Sometimes	30 (18.5%)	4 (2.5%)	34 (21.0%)		
Presence of livestock nearby	Yes	195 (90.7%)	2 (0.9%)	197 (91.6%)	0.004
	No	15 (7.0%)	3 (1.4%)			18 (8.4%)
Contact with a COVID-19 patient	No	185 (89.8%)	13 (6.3%)	198 (96.1%)	2.033	0.436
Yes	7 (3.4%)	1 (0.5%)	8 (3.9%)			

### 3.5 Assessment of associated factors of *SARS-CoV-2* in humans

Mask-wearing in public was significantly associated with *SARS-CoV-2* status in human participants (χ^2^ = 13.617, *p* = 0.009; Table 4). No significant associations were observed for gender, age group, daily contact time with dogs, zoonotic awareness, social distancing, vaccination status, frequency of interaction with dogs, hand washing, prior COVID-19 diagnosis, or concern about zoonotic transmission (*p* > 0.05).

**Table 4:** Association between demographic data and *SARS-CoV-2* occurrence in human.

Variable	Categories	SARS-CoV-2		Total	Chi-square value	*p*-value
		Negative	Positive			
Gender	Male	1 (0.9%)	72 (61.5%)	73 (62.4%)	0.00	1.000
	Female	0 (0%)	44 (37.6%)	44 (37.6%)		
Age group	18–24	0 (0%)	73 (62.4%)	73 (62.4%)	3.061	0.548
	25–34	1 (0.9%)	28 (23.9%)	29 (24.8%)		
	35–44	0 (0.0%)	5 (4.3%)	5 (4.3%)		
	45–54	0 (0.0%)	6 (5.1%)	6 (5.1%)		
	55 & above	0 (0.0%)	4 (3.4%)	4 (3.4%)		
Average time with dog in a day	< 1 h	0 (0.0%)	49 (43.0%)	49 (43.0%)	2.018	0.365
	1–3 h	0 (0.0%)	27 (23.7%)	27 (23.7%)		
	>3 h	1 (0.9%)	37 (32.5%)	38 (33.4%)		
Zoonotic awareness	No	0 (0.0%)	7 (6.0%)	7 (6.0%)	0.000	1.00
	Yes	1 (0.9%)	109 (93.2%)	110 (94.1%)		
Social distancing	Always	1 (0.9%)	11 (9.5%)	12 (10.3%)	8.742	0.068
	Never	0 (0.0%)	1 (0.9%)	1 (0.9%)		
	Often	0 (0.0%)	23 (19.8%)	23 (19.8%)		
	Rarely	0 (0.0%)	27 (23.3%)	27 (23.3%)		
	Sometimes	0 (0.0%)	53 (45.7%)	53 (45.7%)		
Received CoV-19 vaccine	No	1 (0.9%)	37 (31.6%)	38 (32.5%)	0.141	0.707
	Yes	0 (0.0%)	79 (67.5%)	79 (67.5)		
Interact with dogs	Always	1 (0.9%)	34 (29.3%)	35 (30.2%)	2.334	0.675
	Never	0 (0.0%)	4 (3.4%)	4 (3.4%)		
	Often	0 (0.0%)	31 (26.7%)	31 (26.7%)		
	Rarely	0 (0.0%)	12 (10.3%)	12 (10.3%)		
	Sometimes	0 (0.0%)	34 (29.3%)	34 (29.3%)		
Wear mask in public	Always	0 (0.0%)	2 (1.7%)	2 (1.7%)	13.617	0.009
	Never	0 (0.0%)	5 (4.3%)	5 (4.3%)		
	Often	1 (0.9%)	7 (6.0%)	8 (6.9%)		
	Rarely	0 (0.0%)	49 (42.2%)	49 (42.2%)		
	Sometimes	0 (0.0%)	52 (44.8%)	52 (44.8%)		
Hand washing	Always	1 (0.9%)	60 (51.7%)	61 (52.6%)	0.909	0.923
	Never	0 (0.0%)	1 (0.9%)	1 (0.9%)		
	Often	0 (0.0%)	28 (24.1%)	28 (24.1%)		
	Rarely	0 (0.0%)	6 (5.2%)	6 (5.2%)		
	Sometimes	0 (0.0%)	20 (17.2%)	20 (17.2%)		
Diagnosed with COVID-19	No	1 (0.9%)	105 (92.9%)	106 (93.8%)	0.000	1.000
	Yes	0 (0.0%)	7 (6.2%)	7 (6.2%)		
Concern with CoV-19 Zoonotic	No	0 (0.0%)	16 (14.2%)	16 (14.2%)	0.000	1.000
	Yes	1 (0.9%)	96 (85.0%)	97 (85.9%)		

## 4 Discussion

This study provides evidence of SARS-CoV-2 exposure and infection in dogs in Ghana, compatible with human-to-animal transmission. It aligns with evidence from other researchers on the possibility of *SARS-CoV-2* infections in animals (16). The detection of *SARS-CoV-2* in dogs through both PCR (2.3%) and serological testing (6.5%) demonstrates that companion animals in these regions are susceptible to natural infection. These findings are consistent with global observations from North America, Europe, and Asia, where sporadic cases of infection have been reported in dogs living with COVID-19-positive patients (17, 18). Although the low prevalence in our study may reflect the small sample size and possibly reduced rates of human COVID-19 during the sampling period, it nonetheless clearly confirms that such transmissions do occur in Ghana.

The seropositivity observed in humans, 99.1% (116/117) in this study, is a remarkable result, highlighting the widespread community transmission of *SARS-CoV-2* that had already occurred at the time this study was done; it also highlights that the majority of the population has been vaccinated against COVID-19 disease. This high rate of seroprevalence aligns with findings from serological surveys in other countries after significant pandemic waves, indicating extensive exposure within the population or vaccine-induced immunity since some of the participants admit receiving one or more doses of *SARS-CoV-2* vaccination (19, 20). To differentiate participants who tested positive for structural protein, indicating field exposures, from those with vaccine-induced antibodies, NCP ELISA was used. Conversely, the low PCR positivity rate in humans (5.1%) suggests that the study period did not coincide with a major acute outbreak, and most participants were likely either previously exposed or had recovered.

Among the primary objectives of this study was to elucidate risk factors associated with *SARS-CoV-2* infection in both human and canine populations. In humans, statistical analysis revealed no significant association between PCR positivity (active infection) and demographic variables such as gender, age, profession, vaccination status, and majority of behavioral practices such as duration of contact with dogs, adherence to social distancing, and hand hygiene. However, in agreement with extensive global research findings, our study identified a strong, statistically significant association between infrequent mask usage in public and a positive *SARS-CoV-2* active infection (*p-value* = 0.009). The data indicated that individuals who tested positive were predominantly those who “rarely” or “sometimes” wore masks, with no positive cases among those who “always” wore masks. This identifies inconsistent mask-wearing as a key behavioral risk factor for *SARS-CoV-2* infection. This result is consistent with global research affirming that face masks are a critical intervention for curbing the spread of *SARS-CoV-2* by blocking respiratory droplets (21, 22). The finding again supports population-level studies showing that communities with high mask adherence had lower COVID-19 rates (23). Thus, the elevated infection risk among those who did not consistently wear masks highlights the vital role of this measure in personal protection during the pandemic (24).

This study found no statistically significant association between gender, age, Profession, Zoonotic Awareness, Social Distancing, Receipt of the COVID-19 vaccine, and Hand Washing, except for wearing a mask in public. In conformity with the findings, some studies also did not establish any significant associations between sex and *SARS-CoV-2* infection risk regardless of the outcome or severity of the disease (25). However, other systematic reviews and meta-analyses have reported male sex as a risk factor for severe COVID-19 outcomes and mortality (26). Age interestingly did not also show any association with *SARS-CoV-2* infection risk as demonstrated by numerous global studies as a critical demographic factor, with older individuals (those over 60 or 75 years) consistently cited as being at higher risk for both contracting *SARS-CoV-2* and developing severe or mortality (26–28), while children are generally reported to have lower susceptibility to clinical infection and having less severe cases (25).

This study did not establish any association of *SARS-CoV-2* infection rates in the professional population, thus veterinary students, veterinarians, para-veterinarians, dog owners, and dog breeders; this finding contradicts other studies that have indeed documented that certain occupations, especially frontline and healthcare workers, are at high risk of *SARS-CoV-2* infection due to higher exposure levels (27, 29).

The study indicated that awareness of zoonotic diseases was high among participants, but there was no association established. In the same vein, our research indicates no significant association between adherence to social distancing and *SARS-CoV-2* active infection in human participants, with a *p-value* of 0.068. This contrasts with global findings where social distancing has been widely recognized as an effective non-pharmaceutical intervention to reduce *SARS-CoV-2* transmission (30). Studies have shown that social distancing measures can significantly curtail the daily case growth rate and mortality rate of COVID-19 (31). The absence of a significant association in our study might be influenced by various factors, including the adherence levels, or the prevalence of the virus during the sampling period.

This study again found no significant relation between vaccination status and *SARS-CoV-2* active infection and exposure (*p-value* = 0.707). This is a significant finding given the strong evidence globally supporting the effectiveness of COVID-19 vaccines in preventing infection and severe disease (32–36). Studies have shown that vaccinated people, particularly those who have gotten booster doses, have significantly lower rates of SARS-CoV-2 infection than people who have not received the vaccine (37, 38). Our research did not establish any significant association between hand washing practices and *SARS-CoV-2* active infection as established by other researchers (30).

Among the canine cohort, a particularly noteworthy finding was the strong positive association observed between active *SARS-CoV-2* infection in dogs and close proximity to livestock (*p* = 0.004). This statistically significant association suggests a potential epidemiological linkage that needs further investigation.

Our study did not establish age, sex, breed, management system, and vaccination status, among others, as significant factors associated with *SARS-CoV-2* infection in dogs. However, some researchers suggested that age can play a role, as was established by Nilsson et al. (39), that younger animals were more likely to be infected with *SARS-CoV-2* than older dogs. Other researchers also reported a higher prevalence of *SARS-CoV-2* in dogs under 1 year of age (40). Similarly, with regard to sex, a study conducted in Romania on *SARS-CoV-2* infection and exposure in cats and dogs found no significant association with sex (41). On the contrary, Sulehria et al. (40) demonstrated a higher prevalence of *SARS-CoV-2* infection in male dogs compared to females, while another group of scientists reported a higher prevalence of *SARS-CoV-2* in female dogs (42). Contrary to our findings, where management system of dogs did not influence dogs' exposure to SARS-CoV-2 a study suggested an intensive system of housing dogs, which reduces a dog's tendency to roam as a risk factor due to pets spending more time indoors and thus having closer contact with an infected owner (43). This highlights that while the management system itself might not be a direct risk factor, the resulting human-animal interaction patterns are crucial.

As identified from this study, vaccination status had no significant association with a dog's routine vaccination status (core vaccinations) and *SARS-CoV-2* infection. It is necessary to note that specific vaccines for canine *SARS-CoV-2* are not widely used or explicitly mentioned as a factor in most published studies on natural infection, as canine susceptibility is generally low and infections are often mild (44, 45).

Although dogs are predominantly infected via human-to-animal transmission, this result raises the hypothesis of possible reverse zoonosis from humans to livestock, followed by secondary transmission to dogs, or exposure to a shared environmental reservoir (46). These observations are consistent with reports highlighting the complexity of multi-species transmission dynamics in settings characterized by high levels of human-animal interface (47) and environments where diverse animal species cohabit and interact (8). In this study however, no livestock (cattle, sheep, goats, or others) were sampled in this study. Therefore, inference regarding livestock as a source or reservoir is a plausible situation which requires dedicated investigation that includes direct sampling of livestock.

In contrast to our initial hypothesis, statistical analysis did not demonstrate a relevant association between canine *SARS-CoV-2* infection status and exposure to people with confirmed human COVID-19 cases. This lack of association may result from several factors, including potential underreporting of human infections, the occurrence of asymptomatic or subclinical cases among owners, or indirect exposure via unidentified infected individuals in the community. These results are congruent with those of Bienzle et al. (10), who similarly reported that, although human-to-animal transmission of *SARS-CoV-2* has been recorded, it does not consistently correspond with recorded contact histories. The high virus transmissibility and the existence of several, possibly unidentified, exposure channels within the human-animal interface could be the causes of this disparity.

The evaluation of multiple additional variables, including dog breed, management practices, core vaccination status, and dietary factors, did not identify any significant associations with *SARS-CoV-2* infection in the canine population. This indicates that, within this cohort, the principal determinant of infection risk is likely the level of viral prevalence in the surrounding human community, rather than specific husbandry methods or genetic predisposition. These findings are consistent with current scientific consensus, which holds that canine susceptibility to *SARS-CoV-2* is broad and that infection occurs opportunistically, primarily as a result of exposure to infected humans (48).

The elevated seropositivity observed among veterinary professionals and dog owners highlights their increased risk of exposure, likely attributable to regular and close interactions with both animals and the public. Nevertheless, the finding that their infection rates did not significantly differ from the overall community prevalence indicates that occupational exposure to dogs did not constitute a major independent risk factor for *SARS-CoV-2* infection beyond the risk associated with community transmission.

This study provides evidence of *SARS-CoV-2* transmission from humans to dogs in Ghana and identifies close interaction with livestock as a potential new risk factor. The exceptionally high seroprevalence among humans indicates extensive previous exposure within the population. Collectively, these results emphasize the multi-host nature of *SARS-CoV-2* and underscore the necessity of applying a “One Health” framework to monitor the virus across human, animal, and environmental interfaces. Further research employing longitudinal study designs and expanded sample sizes is warranted to clarify the transmission dynamics among humans, livestock, and companion animals.

## 5 Limitations

This study was not without limitations. In this study, only five dogs were positive by RT-PCR, and this small number of positive cases substantially limits the statistical power of the canine risk-factor analyses and increases the risk of unstable estimates; associations involving canine infection should therefore be interpreted with caution. In addition, the cross-sectional design does not allow the direction of transmission or temporal relationships to be established. Another point is that purposive sampling strategy was used I this study, which maximized detection among higher-risk groups but limits the representativeness of the findings and their generalizability to the wider population. In addition, no livestock were sampled in this study, therefore associations between canine infection and proximity to livestock could not be mechanistically examined. Finally, viral sequencing was not performed in this study, therefore circulating variants could not be identified and phylogenetic relationships could not be assessed.

## 6 Conclusion

This study provides evidence of natural SARS-CoV-2 infection in dogs in Ghana, indicating potential human-driven spillover to companion animals within the study area. The detection of viral RNA and specific antibodies confirms canine susceptibility under natural conditions, consistent with global observations; however, because the study was cross-sectional and lacked viral sequencing and phylogenetic analysis, the direction of transmission cannot be established, and these results should be interpreted as evidence of exposure rather than confirmed transmission. Infection was not significantly associated with conventional demographic, husbandry, or behavioral variables, and although dogs are unlikely to sustain the pandemic, their infection status may serve as a useful indicator of community-level human transmission, underscoring the value of a “One Health” approach to monitoring the virus across human, animal, and environmental interfaces.

Further research employing longitudinal designs, larger sample sizes, and sequencing of PCR-positive samples is needed to clarify transmission dynamics and identify circulating variants among humans, livestock, and companion animals. Public health and veterinary authorities should establish integrated surveillance systems covering companion animals and livestock to enable timely detection of cross-species transmission, while veterinarians should consider household COVID-19 history when assessing animals with respiratory or systemic signs. Awareness should be strengthened among veterinary professionals, dog owners, breeders, and farmers regarding the potential for human-animal SARS-CoV-2 transmission, and policymakers should endorse and resource a “One Health” strategy for pandemic preparedness that promotes coordinated action and public education on the zoonotic risks associated with COVID-19.

## Data Availability

The datasets generated and analysed during this study are available in the Zenodo repository, https://doi.org/10.5281/zenodo.21365605. Further enquiries can be directed to the corresponding author.
